# Outbreak of yellow fever in central and southwestern Uganda, February–may 2016

**DOI:** 10.1186/s12879-018-3440-y

**Published:** 2018-11-03

**Authors:** Leocadia Kwagonza, Ben Masiira, Henry Kyobe-Bosa, Daniel Kadobera, Emily B. Atuheire, Bernard Lubwama, Atek Kagirita, Edson Katushabe, John T. Kayiwa, Julius J. Lutwama, Joseph C. Ojwang, Issa Makumbi, Alex Riolexus Ario, Jeff Borchert, Bao-Ping Zhu

**Affiliations:** 1 Uganda Public Health Fellowship Program, P.O. Box 7272, Kampala, Uganda; 2 Ministry of Health of Uganda, Kampala, Uganda; 3 Makerere University school of Public Health, Kampala, Uganda; 4 World Health Organization, Uganda Country Office, Kampala, Uganda; 5 Uganda Virus Research Institute, Entebbe, Uganda; 6 United States Centers for Disease Control and Prevention, Kampala, Uganda; 7 Division of Global Health Protection, Center for Global Health, United States Centers for Disease Control and Prevention, Atlanta, GA USA

**Keywords:** Yellow fever, Outbreak investigation, Uganda

## Abstract

**Background:**

On 28 March, 2016, the Ministry of Health received a report on three deaths from an unknown disease characterized by fever, jaundice, and hemorrhage which occurred within a one-month period in the same family in central Uganda. We started an investigation to determine its nature and scope, identify risk factors, and to recommend eventually control measures for future prevention.

**Methods:**

We defined a probable case as onset of unexplained fever plus ≥1 of the following unexplained symptoms: jaundice, unexplained bleeding, or liver function abnormalities. A confirmed case was a probable case with IgM or PCR positivity for yellow fever. We reviewed medical records and conducted active community case-finding. In a case-control study, we compared risk factors between case-patients and asymptomatic control-persons, frequency-matched by age, sex, and village. We used multivariate conditional logistic regression to evaluate risk factors. We also conducted entomological studies and environmental assessments.

**Results:**

From February to May, we identified 42 case-persons (35 probable and seven confirmed), of whom 14 (33%) died. The attack rate (AR) was 2.6/100,000 for all affected districts, and highest in Masaka District (AR = 6.0/100,000). Men (AR = 4.0/100,000) were more affected than women (AR = 1.1/100,000) (*p* = 0.00016). Persons aged 30–39 years (AR = 14/100,000) were the most affected. Only 32 case-patients and 128 controls were used in the case control study. Twenty three case-persons (72%) and 32 control-persons (25%) farmed in swampy areas (OR_adj_ = 7.5; 95%CI = 2.3–24); 20 case-patients (63%) and 32 control-persons (25%) who farmed reported presence of monkeys in agriculture fields (OR_adj_ = 3.1, 95%CI = 1.1–8.6); and 20 case-patients (63%) and 35 control-persons (27%) farmed in forest areas (OR_adj_ = 3.2; 95%CI = 0.93–11). No study participants reported yellow fever vaccination. Sylvatic monkeys and *Aedes* mosquitoes were identified in the nearby forest areas.

**Conclusion:**

This yellow fever outbreak was likely sylvatic and transmitted to a susceptible population probably by mosquito bites during farming in forest and swampy areas. A reactive vaccination campaign was conducted in the affected districts after the outbreak. We recommended introduction of yellow fever vaccine into the routine Uganda National Expanded Program on Immunization and enhanced yellow fever surveillance.

## Background

Yellow fever is an acute viral hemorrhagic disease caused by the yellow fever virus, a single-stranded RNA virus that belongs to the genus *Flavivirus* [1, 2]. It is transmitted from humans to humans or from animals to humans by *Aedes* mosquitos [1]. The disease is endemic in tropical areas; in Africa, South America, and Central America [2, 3]. The Global Health Security Agenda (GHSA) encourages all member states to be able to rapidly detect and respond to outbreaks [4].

Yellow fever is considered to be a re-emerging disease due to increasing reports of its occurrence in different parts of the world in the recent years [5]. Worldwide, the number of yellow fever cases has increased over the past 20 years. This might be attributable to multiple factors, including declining population immunity to infection [6], increased human activities such as deforestation, urbanization [7], population movements [8], and climate change [9]. In 2013, the disease affected an estimated 130,000 people and caused about 78,000 deaths in Africa [10]. There is no specific treatment for yellow fever; only supportive treatment is available to manage symptoms. Without treatment, up to 50% of severely affected persons die. An effective yellow fever vaccine is available; a single dose of this vaccine provides life-time protection against the disease [3].

Lying in the so-called “yellow fever belt”, Uganda is one of the 32 African countries at risk of yellow fever transmission [2] . Since the identification of the first outbreak in 1941, several outbreaks have occurred in Uganda [2, 11], the largest of which affected 181 people and resulted in 45 deaths in northern Uganda in 2010 [12].

Since 2000, surveillance for yellow fever in Uganda has been conducted through the Integrated Disease Surveillance and Response (IDSR) strategy [13]. This strategy enables timely detection of and response to outbreaks to prevent further spread. On 26 March 2016, the IDSR focal person in Masaka District, southern Uganda, alerted the Public Health Emergence Operations Center (PHEOC) of the Ministry of Health (MoH) that within a one-month period, three men from the same extended family had died of a “strange disease” with bleeding symptoms. Fearing an outbreak of a viral hemorrhagic fever (VHF), the MoH immediately activated the VHF response plan, established an isolation unit at the Masaka Regional Referral Hospital, and initiated active case-finding. Six blood samples were collected from patients at the isolation unit and tested for Ebola Virus, Marburg Virus, Crimean-Congo Haemorrhagic Fever, and Rift Valley Fever in the Viral Special Pathogen Laboratory at the Uganda Virus Research Institute (UVRI). However, the samples tested negative for all the tested VHFs. Based on the clinical presentation of the patient and the initial laboratory results, the reserved samples were then sent to the Arbovirus laboratory for further testing including yellow fever testing. On 8 April 2016, three samples from Masaka District tested positive for yellow fever by both PCR and IgM antibody tests. On 9 April 2016, MoH declared a yellow fever outbreak and launched an outbreak response. After the declaration, another cluster of cases was reported in Rukungiri District, southwestern Uganda.

As part of the outbreak response, we conducted an epidemiologic investigation to determine the scope of the outbreak, identify risk factors for transmission, and recommend evidence-based measures for outbreak prevention and control.

## Methods

### Case definition and investigation

For this investigation, we defined a suspected case as onset of unexplained fever (negative for malaria rapid diagnostic test, non-responsive to malaria treatment, and not explained by any other known reasons) in a resident or visitor of the affected districts from January to June 2016, plus ≥2 of the following symptoms that are not explained by other causes: abdominal pain, diarrhea and headache. A probable case was onset of unexplained fever, plus ≥1 of the following unexplained symptoms: jaundice, unexplained bleeding, or liver function abnormalities (e.g., elevated levels of transaminases). A confirmed case was a suspected or probable case with positive IgM or PCR tests for yellow fever.

For case-finding, we visited the nine referral health facilities in the areas affected by the outbreak, including five in Masaka, two in Kalungu, one in Kalangala, and one in Rukungiri District, and reviewed out-patient, in-patient and laboratory records to identify potential cases. District health offices announced the outbreak on local radio stations, and urged people with compatible symptoms to seek care at the nearest health centers. Physicians at health centers and hospitals were urged to report any suspicious cases to MoH.

After a suspected case-patient was reported, we conducted case-investigation interviews. We interviewed admitted patients at health facilities. For suspected case-persons who had been discharged or never sought care, we visited the communities to conduct the interviews with the help of Village Health Team members. Suspected case-persons who met the case definition after the case-investigation interviews were referred for admission for clinical, epidemiologic, and laboratory investigations at designated treatment centers. Some case-patients self-referred to the treatment centers after hearing radio announcements.

We asked the case-patients about their potential risk factors for yellow fever, and entered the data into a line list. From the descriptive epidemiologic analyses of the line list, we hypothesized that farming near forested areas or near swampy areas, and presence of monkeys around residence and cultivation areas were risk factors for yellow fever transmission. We also collected data on history of yellow fever vaccination.

### Case-control study

We tested the above hypotheses using a frequency-matched case-control study with a case-to-control ratio of 1:4. Controls were persons from the same village who never had any symptoms resembling yellow fever during January -to June 2016, frequency-matched by sex and age (±5 years). We selected controls using systematic random sampling from a list of households from the same village as the case-persons.

### Laboratory investigations

We could only collect blood samples from 22 of the 42 case-patients. We collected approximately 5 ml of blood from each of 22 case-patients as soon as a person was suspected of having yellow fever, and transported the samples to UVRI for testing. The samples were tested for yellow fever IgM and/or virus. Yellow fever antibodies were identified using the MAC-ELISA assay [14]. PCR was performed to identify yellow fever virus using RNA extraction and the TaqMan assay. Yellow fever virus primers were designed from published GenBank sequences, including the South American, African, and vaccine strains [15]. The TaqMan primers and hydrolysis probe were designed as described by Tamura et al. [16].

### Environmental assessment

We observed areas where case-patients lived and worked to evaluate for the presence of sylvatic monkeys (the reservoir for yellow fever). A team of laboratorians and entomologists from UVRI also conducted entomological assessments for the presence of vectors and breeding sites using light traps, larval sampling, and oviposition traps around the residences and agricultural fields of cases and controls. Entomology teams laid ovitraps in some of the homesteads in the villages where cases were identified. In each village, 25 ovitrap cups were placed in trees, under banana stems, under shrubs, and under rocks. The teams examined the environment around the selected houses and farmland to identify mosquito breeding sites. They conducted indoor and outdoor larval and pupae surveys using a pipette and squeeze bulb or by scooping using a ladle in habitats with standing water. In each village, the team also carried out pyrethrum insecticide (permethrin and imiprothrin) spray catches in five or six houses early in the morning (starting at about 6:00 a.m.) to sample adult endophilic mosquito vectors following guidelines [17]. Additionally, CDC light traps baited with dry ice (solid carbon dioxide) were set in the homesteads and nearby bushes in the evening, starting at 5:00 pm, to collect adult mosquitoes. In each village, five light traps were set up, and trapping was done for two nights.

### Statistical analysis

We performed descriptive epidemiologic analyses to generate hypotheses on risk factors for yellow fever. In analyzing the data from the case-control study, we used multivariable conditional logistic regression to account for the matched case-control study design and to reduce confounding.

## Results

From the retrospective review of health facility records, the outbreak started from February and ended in May 2016. The total number of people evaluated in this outbreak was 42. We identified 35 probable and 7 confirmed case-patients. Nine additional persons were evaluated and their blood samples were taken for testing; however, they did not meet the probable case definition and their blood samples were negative for yellow fever. Our final data analysis excluded these nine persons, and focused on the probable or confirmed cases.

### Symptomology and demographic characteristics

**Fig. 1:**
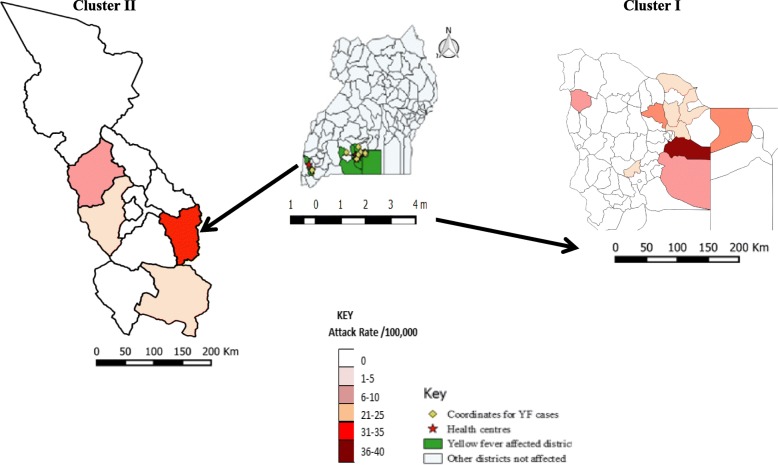
Attack rate of yellow fever by sub-county during an outbreak: Uganda, February – May, 2016

Common symptoms among the 42 probable and confirmed case-patients included fever (100%), jaundice (69%), abdominal pain (60%), and vomiting (55%) (Table 1). The probable and confirmed cases formed two separate geographic clusters in seven districts: Cluster I was located in Districts of Masaka Kalangala, Rakai, Bukomansimbi, Lyantonde and Kalungu in Central Uganda; Cluster II was in Rukungiri District in southwestern Uganda. The overall attack rate in the seven affected districts was 2.6 /100,000. Of the seven districts affected by the outbreak, Masaka had the highest attack rate (6.0/100,000). Of the sub-counties affected, Buwunga Sub-county in Masaka District had the highest attack rate (40/100,000), followed by Kebisoni Sub-county in Rukungiri District (31/100,000) (Fig. 1).

**Table 1:** Distribution of symptoms among yellow fever case-persons during an outbreak: Uganda, February – May, 2016

Symptom	Percent (*n* = 42)
Fever	100
Jaundice	69
Abdominal pain	57
Vomiting	52
Headache	48
Nausea	43
Joint pain	31
Nose bleeding	31
Diarrhea	31
Altered mental state	26
Vomiting blood	26
Muscle pains	21
Gum bleeding	21
Dark stool	14
Hyperemia of the eyes	7.1
Oliguria	4.8

Males had a higher attack rate (4.0/100,000) than females (1.1/100,000). The age range of the probable case-patients was 3–64 (median: 32) years. Of all age groups, persons aged 30–39 years had the highest attack rate (17/100,000) (Table 2). The case fatality rate was 33% (14/42). Case fatality rate among males was 36% (12/33) while as for females was 22% (2/9). The age-group of 20–30 years had the highest case fatality rate of 36% (6/17).

**Table 2:** Attack rate of yellow fever by sex, age and district during an outbreak: Uganda, February–May, 2016

	Case-persons	Population	Attack rate (/100,000)
Overall (seven districts)	42	1,643,235	2.6
Sex			
Male	33	816,529	4.0
Female	9	826,706	1.1
Age (years)			
0- < 10	2	234,366	0.85
10–19	6	178,035	3.4
20–29	17	114,053	15
30–39	12	69,545	17
40–49	2	44,509	4.5
50–59	2	25,732	7.8
60–64	1	9041	11
Masaka District	19	314,823	6.0
Male	15	158,848	9.4
Female	4	155,975	2.6
Kalangala District	3	57,604	5.2
Male	3	32,963	9.1
Female	0	24,641	0
Rukungiri District	11	323,021	3.4
Male	10	161,194	6.2
Female	1	161,827	0.62
Kalungu District	5	184,131	2.7
Male	3	89,362	3.4
Female	2	94,769	2.1
Bukomansimbi District	2	151,075	1.3
Male	0	74,405	0
Female	2	76,670	2.6
Rakai District	1	518,008	0.19
Male	1	253,054	4.0
Female	0	264,954	0
Lyantonde District	1	94,573	1.1
Male	1	46,703	2.1
Female	0	47,870	0

More than half (64%) of the cases were farmers; other occupations represented in case-persons included sand miners (5%), wood colliers (5%), small shop owners (3%), teacher (2%), and casual labor (3%). Of note, all case-persons were engaged in farming activities to at least some extent (Table 3).

**Table 3:** Characteristics of yellow fever case-persons during an outbreak: Uganda, February – May, 2016

Variable	Cluster I^b^	Cluster II^c^
Sex		
Male	23	10
Female	8	1
Age group		
0- < 10	1	1
10–19	6	0
20–29	11	6
30–39	9	3
40–49	2	0
50–59	1	1
60–64	1	0
Occupation		
Peasant farmer	22	5
Sand miner	0	2
Wool collier	1	1
Shopkeeper	1	0
Teacher/student	4	1
Casual labour	3	2
Serology^a^		
Positive	6	1
Negative	8	7
RT PCR^a^		
Positive	5	1
Negative	9	7
Vital Status		
Dead	12	2
Alive	22	8
Case Classification		
Probable	25	10
Confirmed	6	1
Clinical features		
Fever	31	11
Jaundice	19	2
Vomiting	19	2
Headache	23	7
Any bleeding	16	0

^a^ *All samples were tested by RT PCR and serology*

^b^ *Cluster II includes districts of: Masaka, Kalangala, Rakai, Bukomansimbi, Lyantonde and Kalungu*

^c^ *Cluster II includes Rukungiri District*

### Distribution of cases by time

The primary case-person’s onset of symptoms occurred on 14 February 2016. The outbreak lasted until early May. When the epidemic curve was stratified by cluster, Cluster I started in mid-February in Masaka District; Cluster II started at the end of March in Rukungiri District. The outbreak stopped after May 7 (Fig. . 2). Following the declaration of a yellow fever outbreak, MoH planned for mass yellow fever vaccination campaign in the three most affected Districts of Masaka, Kalangala and Rukungiri. MoH distributed 692,700 yellow fever vaccine doses (306,400 for Masaka, 331,100 for Rukungiri and 55,200 for Kalangala Districts) depending on size of the population. The mass vaccination exercise was conducted from 19th to 22nd May 2016 for Masaka and Rukungiri Districts, and on 4th–6th June 2016 for Kalangala District. From this campaign, 627,706 yellow fever vaccine doses were administered achieving administrative vaccination coverage of 94%.

**Fig. 2:**
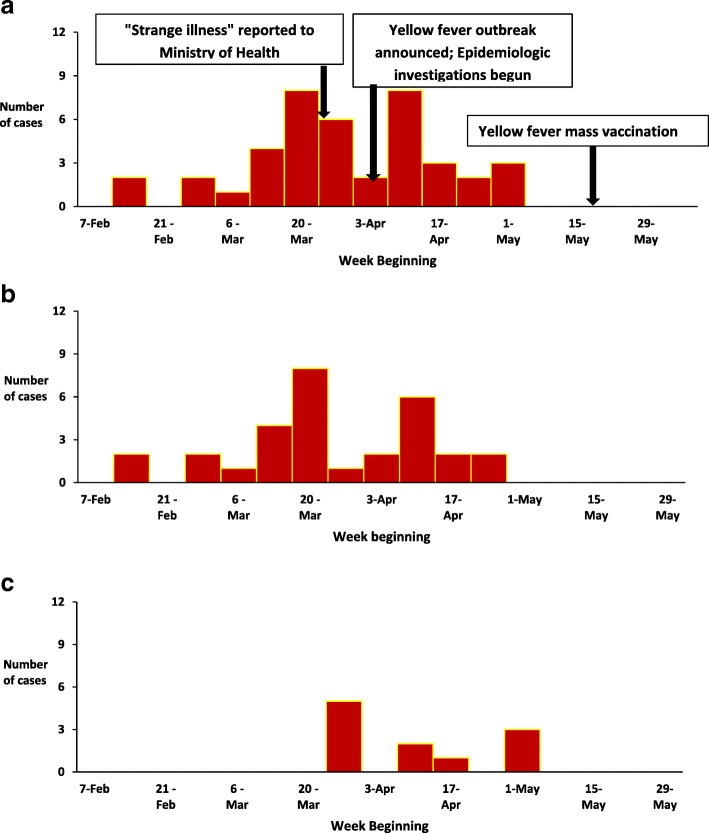
Onset of yellow fever cases by cluster during an outbreak: Uganda, February–May, 2016. **a** Overall epidemic curve. **b** Epidemic curve for Cluster I in southern Uganda. **c** Epidemic curve Cluster II in southwestern Uganda

### Case-control findings

Of the 42 confirmed and probable case-persons, we recruited 32 for our case-control study. Of the 32 cases and 128 controls, 23 (72%) case-persons and 32 (25%) controls had their agriculture crop fields in swampy areas (OR_adj_ = 7.5, 95% CI: 2.3–24); 20 (63%) case-persons and 32 (25%) controls noted the presence of sylvatic monkeys in the fields where they worked (OR_adj_ = 3.1; 95% CI: 1.1–8.8); 20 (63%) case-patients and 35 (27%) controls farmed in the forests (OR_adj_ = 3.2, 95% CI: (0.93–11) (Table 4). When we asked about their vaccination history, no case- or control-person reported a history of vaccination against yellow fever.

**Table 4:** Association between selected exposures and yellow fever during an outbreak: Uganda, February – June, 2016

Variable	% Exposed			
	Cases (*n* = 32)	Controls (*n* = 128)	OR (95% CI)	OR_adj_ (95%CI)
Agriculture fields located in swampy areas	72	25	11 (3.6–33.3)	7.5 (2.3–24)
Presence of monkeys in the agriculture fields	63	25	4.1 (1.7–9.6)	3.1 (1.1–8.8)
Agriculture fields located in forested area	63	27	6.6 (2.3–18.9)	3.2 (0.93–11)

*OR = odds ratio; OR*_*adj*_ *= Odds ratio adjusted for mutual confounding effects of the three variables in the model using conditional logistic regression; CI = confidence interval*

### Findings from environmental assessment

During the environmental assessment, sylvatic monkeys were frequently sighted around the homes and agriculture fields where the case-patients lived or worked. Entomological assessments in the four villages yielded a catch of 3964 mosquitoes (1368 in Kaloddo village, 1386 in Kasaka Village, 650nin Kalinga village and 560 in Bilongo village). In these villages, we identified *Aedes aegypti sp., ae. africanus,* and *Mansonia africana* mosquitoes and numerous breeding sites around farmlands and homesteads. Plastic and metallic containers were found around homes with standing water inside. Many homes had banana plant species known to hold *Ae. simpsoni* larvae in the water in the axils. Numerous larvae of *Ae. africanus* mosquitoes were found in tree holes of *Eucalyptus* sp. trees near homes and in plantations. Full results of the entomological studies during this outbreak and of a risk assessment after the outbreak will be presented in a separate publication.

### Laboratory investigation findings

Yellow fever was confirmed in seven of the 22 blood specimens sent to UVRI, including five positive by PCR and two positive by serology only. Testing of the trapped mosquitos did not yield the yellow fever virus.

## Discussion

Our epidemiologic investigation showed that onset of yellow fever during this outbreak was associated with agriculture activities in and near forests and swampy fields, and with presence of sylvatic monkeys in the agriculture fields. Environmental investigation showed presence of sylvatic monkeys, *Aedes* spp. and other species of mosquitoes involved in yellow fever transmission, and the breeding sites of the mosquitos around farmlands and homesteads of case-patients. The outbreak affected working-age men more than other demographic subgroups, and all case-persons were engaged in some kind of farming activities. Taken together, our investigation indicated that the outbreak probably was sylvatic in nature, and was spread by *Aedes* mosquitoes in the outbreak areas.

Our investigation demonstrated that the outbreak started in Fe bruary, peaked in March and April, and ended in May. In this area, February is the end of the dry season. On the other hand, March through May are the rainy season, during which the mosquito population would greatly increase; at the same time, farming activities such as planting and weeding would also increase, leading to more frequent human exposures to both the vectors (*Adedes* mosquitos) and the reservoir (sylvatic monkeys) for yellow fever in the agriculture field. We trapped and tested several mosquitos in all the areas of probable transmission. However, given the small number of case-patients and their wider geographical distribution, it appears the virus density in the mosquito population was significantly low. This in part explains the negative yellow fever results in the vector population.

Introduction of yellow fever from sylvatic monkeys to the human population has caused many outbreaks in the past [1]. The areas where the current outbreak occurred are undergoing a massive deforestation in favor of agricultural activities [18], resulting in frequent close contact between monkeys and humans. Sylvatic monkeys are now part of the ecosystem in the outbreak areas, especially in Buwunga and Kebisoni Sub-counties, where monkeys were frequently present around homes and in agriculture fields. Anecdotally, we learned that some local residents keep monkeys as pets and consume monkey meat, although we were unable to substantiate these claims. These human-monkey interactions may greatly increase the risk of human exposure to yellow fever and other pathogens.

The stratified epidemic curve in our investigation appeared to suggest that this outbreak might have started in Masaka District, and moved to the other districts. Masaka District is located on a major highway, and the district town is a commercial center where people traveling by the highway would normally stop to shop and dine. These activities might have facilitated the spread of the disease to other districts. Alternately, the outbreaks could have occurred in different districts spontaneously and independently due to transmission of the virus from sylvatic monkeys to humans via mosquito bites. However, these hypotheses could not be differentiated due to lack of genetic sequencing of the viruses from different clusters. In Uganda, the period between March to May falls in the rainy season, during which the mosquito population would increase exponentially, which might partially explain why this outbreak occurred during these months.

In this investigation, we were able to find confirmed (severe) and probable forms of yellow fever infection. However when it comes to seeking healthcare services, as well as in disease outbreaks, it is those severely affected by the disease that either seek care or are detected by the responders in the community. It is therefore possible that the outbreak affected more than what is reported. Michael Johansson estimates that, a severe case of yellow fever may represent an additional three to twenty asymptomatic or mild infections. This therefore indicates that for any detected case, there is a considerable potential for further infections [19] as explained by the iceberg concept.

Early identification, early diagnosis, and prompt response are critical for the successful control of communicable disease outbreaks and for ensuring global health security. The previous yellow fever outbreak in Uganda occurred in 2010 [12]. The outbreak was identified and reported on 8 November 2010, but laboratory confirmation was not completed until 18 December, which was over 40 days after the outbreak was reported. By the end of January 2011, when the outbreak was contained, 181 cases had occurred (including 13 confirmed cases), and 58 persons had died. In comparison, the current outbreak was confirmed 12 days after the outbreak was reported. Consequently, the case count (42 vs. 181) and death toll (14 vs. 58) were reduced by more than 75%. During the six-year time period between 2010 and 2016, the MoH of Uganda under the auspices of the Global Health Security Agenda has substantially enhanced the capacity for outbreak response, the core components of which includes a robust Public Health Emergency Operations Center, enhanced laboratory testing capabilities, a nationwide network of specimen-referral system, and an advanced-level field epidemiology training program. During the current outbreak, all elements of this enhanced outbreak response system worked seamlessly together, which contributed to the shortened response time, reduced morbidity and mortality, and decreased risk of disease spread.

Vaccination is the most important measure for preventing and containing yellow fever outbreaks [3]. During our investigation, none of the study participants reported a history of vaccination against yellow fever. In Uganda, yellow fever vaccination is individually sought by international travellers as a requirement and a few other people who can afford the vaccination cost of $27. In addition, yellow fever vaccination is not part of the routine immunization. This suggests that the population immunity level is low in Uganda. This was a consequence of yellow fever vaccination not being part of routine immunization program in Uganda. As soon as this outbreak was confirmed, the MoH of Uganda mounted a vaccination campaign in and around the outbreak area from May 19, 2016. We believe that interventions such as aggressive case finding and case management, risk communication in affected communities, setting up toll free communication and referral of suspected case among others worked to end this outbreak and effectively prevented the spread of the disease to other areas. Since Uganda has experienced repeated yellow fever outbreaks in the past, adding the yellow fever vaccine to the routine immunization schedule in Uganda may be warranted.

### Study limitations

This study had limitations. First and foremost, genetic sequencing of the viruses found in different districts was not conducted, rendering it impossible to differentiate whether the outbreaks in different districts were the same spreading outbreak or separate outbreaks of sylvatic origin. Secondly, the deceased case-patients were not tested for yellow fever IgM because they died before the response. We conducted proxy interviews with next-of-kin, which could have introduced information bias. Thirdly, self-reporting of exposure history might have resulted in recall bias regarding disease history and risk factors. Sylvatic monkeys on the farmland were reported from the case control study and during the environmental assessment in the outbreak area. However, we were not able to classify the different monkey species seen or reported. Another weakness of this study was that during the outbreak investigation, we had a weak component in animal surveillance. We were not able to test for the presence of yellow fever virus in the monkeys we only observed for their presence. There is also limited evidence of indirect (like fecal testing) method in monkeys for testing yellow virus. Lastly, in the case control study, we used clinical controls; we did not test the control-persons for yellow fever IgM antibodies. This might have introduced bias due to misclassification of control-persons. However, control persons were carefully selected using the case definition above.

### Conclusion and recommendations

This was a yellow fever outbreak that occurred in a susceptible population. The outbreak was associated with farming in forest and swampy areas, which suggested the sylvatic origin. We recommended to the MoH of Uganda to introduce yellow fever vaccination into the routine Uganda National Expanded Program on Immunization.

## Abbreviations

CDC: Centers for Diseases Control and Prevention
CI: Confidence Intervals
IDSR: Integrated Disease Surveillance and Response
MoH: Ministry of Health
OR: Odds Ratio
UVRI: Uganda Virus Research Institute
VHF: Viral Hemorrhagic Fever
WHO: World Health Organization
